# Functional capacity of natural killer cells in HTLV-1 associated myelopathy/tropical spastic paraparesis (HAM/TSP) patients

**DOI:** 10.1186/s12879-019-4032-1

**Published:** 2019-05-17

**Authors:** Gabriel Andrade Nonato Queiroz, Rita Elizabeth Moreira Mascarenhas, Vincent Vieillard, Raphaela Lisboa Andrade, Bernardo Galvão-Castro, Maria Fernanda Rios Grassi

**Affiliations:** 1 Laboratório Avançado de Saúde Pública, Instituto Gonçalo Muniz, Fundação Oswaldo Cruz - Fiocruz, Salvador, Bahia Brazil; 2 Escola Bahiana de Medicina e Saúde Pública, Salvador, Bahia Brazil; 3 Centre d’Immunologie et des Maladies Infectieuses (CIMI-Paris), Sorbonne Universités, UPMC Univ Paris 06, INSERM U1135, CNRS ERL8255, Paris, France

**Keywords:** NK cells, NKp30, Natural cytotoxicity receptor, CD107, HTLV-1, HAM/TSP

## Abstract

**Background:**

Natural killer (NK) cells are part of the innate immune system and provide surveillance against viruses and cancers. The ability of NK cells to kill virus-infected cells depends on the *balance* between the effects of inhibitory and activating *NK cell* receptors. This study aimed to investigate the phenotypic profile and the functional capacity of NK cells in the context of HTLV-1 infection.

**Methods:**

This cross-sectional study sequentially recruited HTLV-1 infected individuals with HTLV-1 associated myelopathy/tropical spastic paraparesis (HAM/TSP) and asymptomatic HTLV-1 (AS) from the Integrated and Multidisciplinary HTLV Center in Salvador, Brazil. Blood samples from healthy blood donors served as controls. NK cell surface receptors (NKG2D, KIR2DL2/KIR2DL3, NKp30, NKG2A, NKp46, TIM-3 and PD-1), intracellular cytolytic (Granzyme B, perforin) and functional markers (CD107a for degranulation, IFN-γ) were assayed by flow cytometry in the presence or absence of standard K562 target cells. In addition, cytotoxicity assays were performed in the presence or absence of anti-NKp30.

**Results:**

The frequency of NKp30^+^ NK cells was significantly decreased in HAM/TSP patients [58%, Interquartile Range (IQR) 30–61] compared to controls (73%, IQR 54–79, *p* = 0.04). The production of cytolytic (perforin, granzyme B) and functional markers (CD107a and IFN-γ) was higher in unstimulated NK cells from HAM/TSP and AS patients compared to controls. By contrast, stimulation with K562 target cells did not alter the frequency of CD107a^+^ NK cells in HAM/TSP subjects compared to the other groups. Blockage of the NKp30 receptor was shown to decrease cytotoxic activity (CD107a) and IFN-γ expression only in asymptomatic HTLV-1-infected individuals.

**Conclusions:**

NK cells from individuals with a diagnosis of HAM/TSP present decreased expression of the activating receptor NKp30, in addition to elevated degranulation activity that remained unaffected after blocking the NKp30 receptor.

## Background

Human T-lymphotropic virus type 1 (HTLV-1) has been associated with adult T-cell leukemia/Lymphoma (ATLL) [1], infective dermatitis [2] and other inflammatory diseases [3]. This virus may also lead to HTLV-1 associated myelopathy/tropical spastic paraparesis (HAM/TSP), a progressive *inflammatory demyelinating disease* affecting the spinal *cord* [4]. Patients with HAM/TSP present an infiltrate of infected T-lymphocytes and cytotoxic T-lymphocytes (CTL) specific for viral antigens in their cerebrospinal fluid, in addition to increased proinflammatory cytokine (IFN-γ, TNF-α) and chemokine (CXCL-9 and CXCL-10) production [5, 6].

High proviral loads have been associated with the development of HAM/TSP [7, 8], as well as with the development of infective dermatitis [9], Keratoconjunctivitis sicca [10] and bronchiectasis [3]. Moreover, increased proviral loads and an exacerbated activation of the immune system may also be seen in asymptomatic individuals infected with HTLV-1 [11, 12].

Proviral load can become suppressed or be maintained at stable levels due to the intense and specific activity of cytotoxic CD8^+^ T-lymphocytes (CTL) against HTLV-1-infected cells [13, 14]. In contrast to CTLs, NK cells are understood to provide surveillance in the defense against viruses and tumor cells, without the need for prior sensitization. NK cell activity is regulated by a dynamic balance of signaling among a vast network of activating and inhibitory receptors, which become triggered upon interaction with their cognate ligands to detect cellular targets while sparing normal cells. Under typical physiological circumstances, NK cells express inhibitory receptors that recognize self-molecules of the HLA-I repertoire, which are constitutively expressed on the surfaces of host cells. In order for NK cells to mount an efficient response, a critical signaling threshold must be reached in which activating receptors exceed the counterbalancing influence of inhibitory receptors [15]. Lower frequencies of circulating NK cells have been reported in patients with HAM/TSP compared to asymptomatic carriers [16–18]. Nonetheless, the role of the NK cellular response in HTLV-1 infection requires further clarification. Accordingly, the present study aimed to investigate the phenotypic profile of NK cells and to evaluate their functional capacity in the context of HTLV-1 infection, especially in subjects with HAM/TSP.

## Methods

### Ethical considerations

The present research protocol was approved by the Institutional Research Board (IRB) of the Bahiana School of Medicine and Public Health (EBMSP) in Salvador, Bahia-Brazil (protocol no. 187/2011). All procedures were performed in accordance with the principles established in the Declaration of Helsinki and its subsequent revisions.

### Patients

For this cross-sectional study, HTLV-1-infected individuals were selected by convenience sampling at the Integrated and Multidisciplinary HTLV Center, (Salvador, Bahia-Brazil). All participants were sequentially included at the time of their previously scheduled appointments. Inclusion criteria were individuals of both genders, 18 to 65 years of age, with an available neurological evaluation used to differentiate asymptomatic from HAM/TSP individuals. Myelopathic symptoms, serological findings, and/or the detection of HTLV-1 DNA, as well as the exclusion of other disorders were all used as indicators in the diagnosis of HAM/TSP [19]. Asymptomatic individuals (AS) were included if their neurological examinations were normal and they reported no clinical complaints. Eighteen laboratory staff and/or healthy blood donors were included as non-infected controls. Any individuals with HIV, HBV and/or HCV were excluded. HTLV-1 infection was diagnosed using ELISA (Cambridge Biotech Corp., Worcester, MA) and confirmed by Western Blot analysis (HTLV blot 2.4, Genelab, Singapore).

### Cells

Peripheral blood mononuclear cells (PBMC) from HTLV-1-infected individuals and non-infected controls were obtained by Ficoll-Hypaque density gradient centrifugation (Sigma Chemical Co., St. Louis, MO) and stored in liquid nitrogen until use. After thawing, any samples presenting less than 85% viability were discarded.

### Immunophenotyping by flow cytometry

**Fig. 1:**
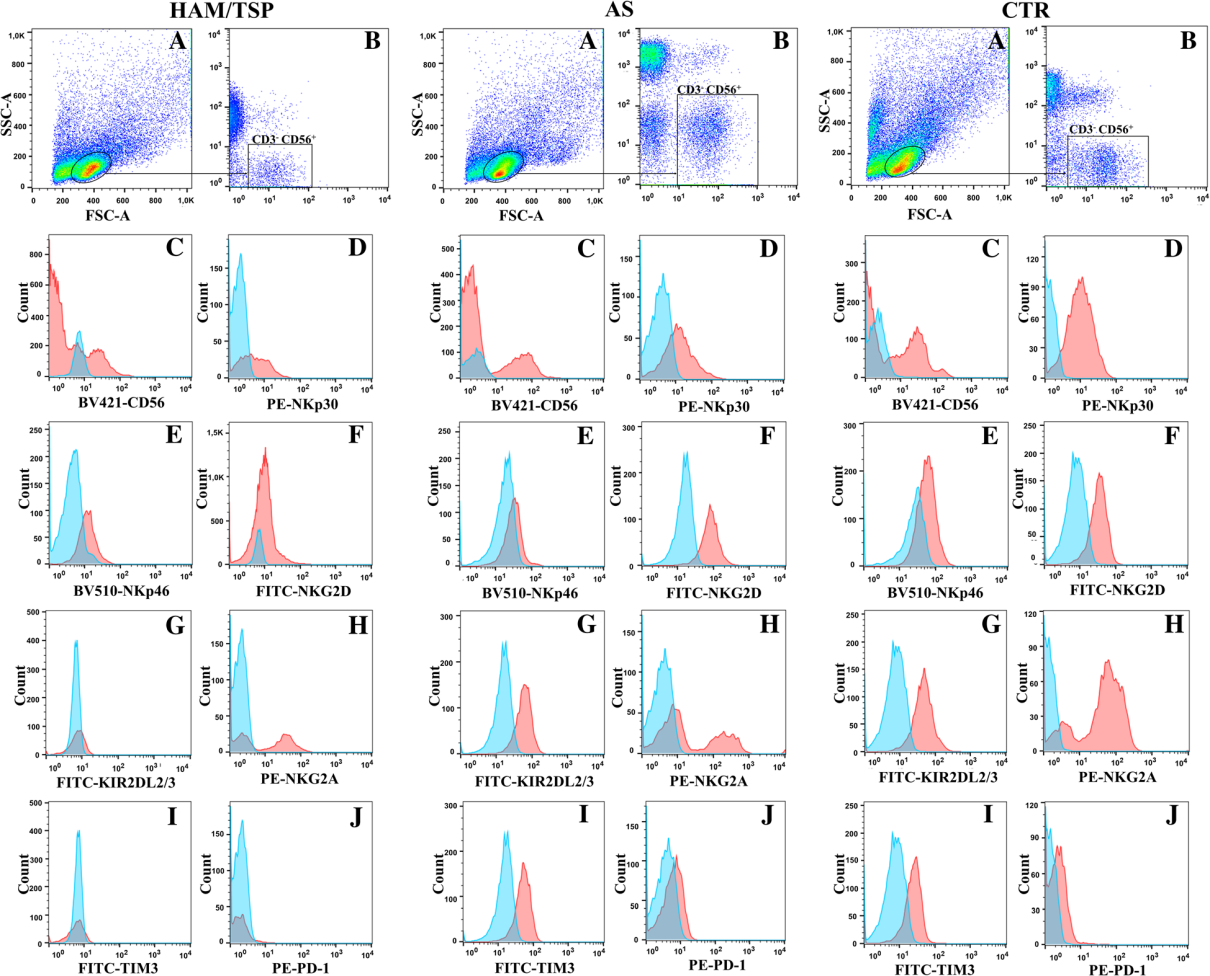
Phenotypic characteristics of NK cells from individuals with HTLV-1 associated myelopathy /Tropical spastic paraparesis (HAM/TSP), asymptomatic HTLV-1-infected individuals (AS) and uninfected controls (CTR). (A) Lymphocyte gate. (B) Selected NK cells (CD3^−^CD56^+^). (C) Expression of CD56. Activating/inhibitory receptors (D-H) and exhaustion markers (I-J) from one representative individual from each group, out of 17 independent experiments performed. Pink histograms represent specific–stained population and blue histograms represent background fluorescence observed with matched isotype control

PBMCs were incubated for 20 min at room temperature with the following combinations of fluorescence-conjugated monoclonal antibodies (MAbs): (FITC)–labeled fluorescein isothiocyanate, anti-NKG2D, anti-KIR2DL2/KIR2DL3 and anti-TIM-3; phycoerythrin (PE)–labeled anti-NKP30, anti-NKG2A and anti-PD-1; Allophycocyanin-cyanin-7 (APCCY7) anti-CD3; Brilliant violet 421 (BV421)-labeled anti-CD56; Brilliant violet 510 (BV510) anti-NKP46. The following isotype controls were used: (APCCY7-IgG2a); (PECY7-IgG1); (BV421-IgG1); (PE-IgG1-extracellular); (PE-IgG1-intracellular); (FITC-IgG1); (BV510-IgG1); (AF647-IgG1). All MAbs were purchased with the (Biolegend, San Diego, CA, EUA), except anti-NKG2A (Miltenyi Biotec, Bergisch Gladbach, Germany). All cells were then washed and fixed in PBS containing 1% formaldehyde (Sigma-Aldrich) for 20 min. Cells were acquired using flow cytometry (BD Facs RSFortessa™, San Jose, CA, EUA) and analyzed by Software FlowJo (Tree Star), with at least 50,000 events considered. Representative flow cytometry dot plots are shown in Fig. 1 (A-I).

### NK cell degranulation assays and intracellular cytokine production

Polyfunctional assays simultaneously detected NK cell degranulation (evidenced by CD107a surface expression) and the intracellular production of IFNγ. 106 PBMC/ml were incubated in RPMI 1640 (Sigma) containing 10% FCS (Gibco, Waltham, MA, USA), 2 mM L-glutamine, 1% nonessential amino acids, 1 mM sodium pyruvate, 100 U/ml penicillin and 100 g/ml streptomycin (Sigma) in the presence of an anti-CD107a-FITC antibody (Biolegend, San Diego, CA, EUA) for 6 h at 37 °C under 5% CO2. After the first hour of culturing, brefeldin A and monensin were added (2 μg/ml) (Sigma). Similar assays were performed in the presence of K562 target cells (ATCC CCL-243) at an effector to target ratio of 1:1 in the presence or absence of 4 μg/ml of anti-NKp30 (Biolegend- San Diego, CA, USA). All cells were then washed and stained with CD3-APC-CY7, CD8-PE-CY7 and CD56-BV421 antibodies (Biolegend - San Diego, CA, USA) for 20 min. After washing, cells were fixed in 1% PBS/formaldehyde for 20 min, followed by two washing cycles with 0.1% PBS-BSA/Saponin. The cells were then incubated with anti-granzyme B-Alexa-Fluor 647 (Becton Dickinson Pharmingen, San Jose, CA, EUA), anti-perforin-PE and anti-IFN-γ-PE (Biolegend, San Diego, CA, EUA) for 30 min. Finally, the stained cells were acquired using flow cytometry (BD Facs RSFortessa™, San Jose, CA, EUA) and analyzed by Software FlowJo (Tree Star) considering at least 50,000 events.

### Proviral load

DNA was extracted from PBMCs using a spin column DNA extraction system (Qiagen, Hilden, Germany). HTLV-1 proviral load was quantified using a previously described real-time TaqMan polymerase chain reaction (PCR) method [20]. HTLV-1 proviral load was calculated as [(average number of HTLV-1 copies)/(average number of albumin copies)] × 2 × 10^6^, and is expressed as the number of HTLV-1 copies per 10^6^ PBMCs.

### Statistical analysis

Age is expressed as mean with standard deviation, while other data are expressed as median and interquartile range (25th and 75th percentiles). Comparisons of proviral load between the AS and HAM/TSP groups were performed using the Mann-Whitney U-test. The Kruskal–Wallis analysis of variance and Bonferroni-Dunn multiple comparison tests were used to compare among healthy donors, AS and HAM/TSP groups. Chi-square test was used to compare sex frequencies. Wilcoxon’s test was used to compare the cytotoxic activity of NK cells in the presence or absence of the anti-NKp30 monoclonal antibody. Correlations were performed using Spearman’s correlation test. Differences were considered significant when *p* < 0.05. GraphPad Prism v5 (La Jolla, CA) software was used for all statistical analyses.

## Results

### Clinical characteristics

A total of 20 individuals with a diagnosis of HAM/TSP, 28 asymptomatic carriers and 18 uninfected healthy controls were included. The mean ages of the HAM/TSP (48.7 ± 10), AS (42.9 ± 11.4) and uninfected control (CTR) (41.4 ± 14.2) groups were similar. The median HTLV-1 proviral load in the HAM/TSP group was significantly higher than the AS group (173,146vs. 10,101copies/10^6^ PBMCs, respectively) (*p* = 0.0001). Spasticity in the lower limbs was present in all HAM/TSP individuals, yet absent in AS and controls. Overactive bladder was detected only in 76% of HAM/TSP individuals (Table 1).

**Table 1:** Clinical and demographic characteristics of all individuals included in the study

Groups	Age (Years) Mean ± SD	Gender - n (%)		PVL	Spasticity in lower limbs	Overactive bladder
		F	M	Median (IQR 25–75)	*n* (%)	*n* (%)
HAM/TSP (*n* = 20)	48.7 ± 10	13 (65.0)	7 (35.0)	173,146 (94,232-240,393)*	20 (100)	13 (76)^a^
AS (*n* = 28)	42.9 ± 11.4	19 (67.9)	9 (32.1)	10,101 (1659-36,326)	0	0
CTR (n = 18)	41.4 ± 14.2	10 (55.6)	8 (44.4)	–	0	0

*HAM/TSP* HTLV-1-associated myelopathy/Tropical spastic paraparesis, *AS* Asymptomatic, *CTR* Uninfected controls, *n* Number, *SD* Standard Deviation, *F* Female, *M* Male, *IQR* Interquartile range, *PVL* HTLV-1 Proviral load (copies/10^6^ PBMC), performed for 14 patients with HAM/TSP and 24 asymtomatic individuals. ^a^Data available from only 17 HAM/TSP patients. Statistical analysis of differences between groups determined by the Kruskal-Wallis test, followed by Dunn’s post-test. Statistical analysis of proviral load performed using the Mann-Whitney test. *(*p* = 0.0001)

Phenotypic profile of inhibitory, activating and exhaustion markers in NK cells.

**Fig. 2:**
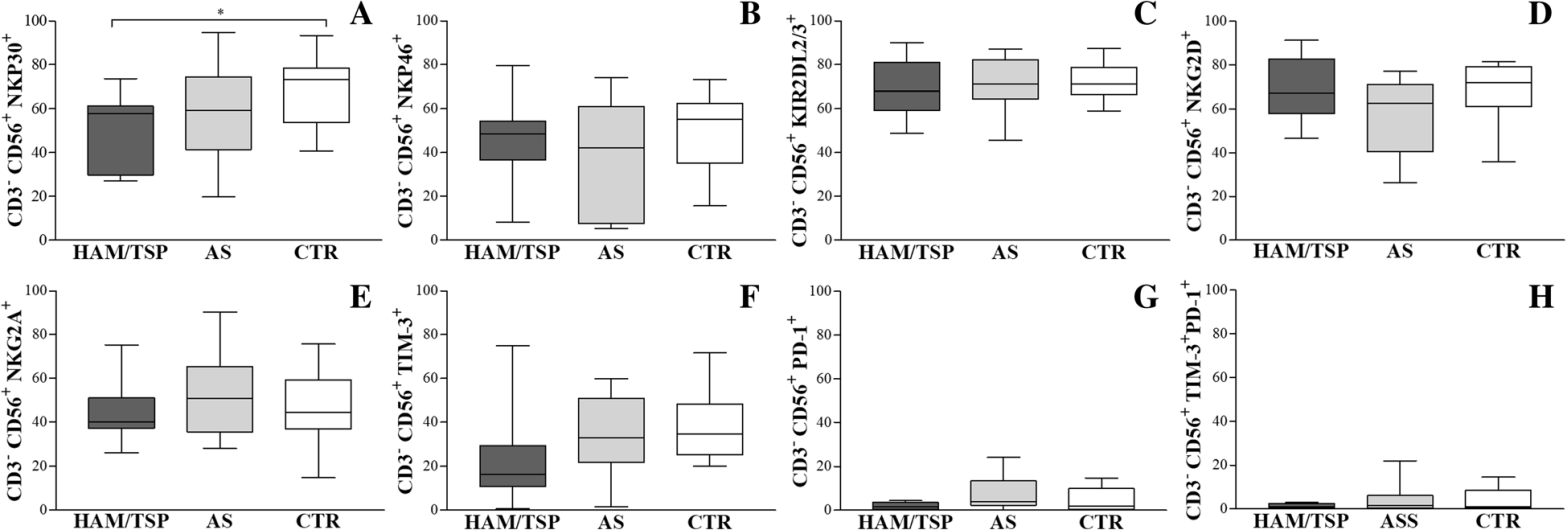
Analysis of cell-surface receptors. HAM/TSP: HTLV-1 associated myelopathy /Tropical spastic paraparesis, *n* = 12; AS: asymptomatic HTLV-1 carriers, *n* = 18. CTR: uninfected controls, *n* = 15. Activating receptors: **a** NKp30, (**b**) NKp46 and (**d**) NKG2D. Inhibitory receptors: **c** KIR2DL2/KIR2DL3 and (**e**) NKG2A exhaustion markers: **f**-**h** TIM-3 and PD-1. Data are expressed as medians and interquartile interval range. Differences were considered significant when *P* ≤ 0.05. Kruskal-Wallis test, followed by Dunn’s post-test

The median frequency CD3^−^CD56^+^ NK cells was similar in HAM/TSP patients (3%, IQR 1–6), AS carriers (2%, IQR 1–3) and CTR (5%, IQR 2–8). In order to better characterize NK cells, cell-surface receptors were extensively analyzed. The frequency of CD56^+^ NK cells in HTLV-1-infected individuals was statistically matched to that of the CTR in terms of inhibitory KIR2DL2/KIR2DL3 and NKG2A receptors, NKG2D and NKp46 activating receptors, as well as markers of cell exhaustion (TIM-3 and PD-1) (Fig. 1 and Fig. 2b-h). In contrast, the frequency of NKp30+ NK cells in individuals with HAM/TSP (58%, [30–61]) was lower compared to CTR (73%, [54–79], (*p* = 0.04) (Fig. 2a).

### Cytotoxic marker and IFN-γ expression by NK cells

**Fig. 3:**
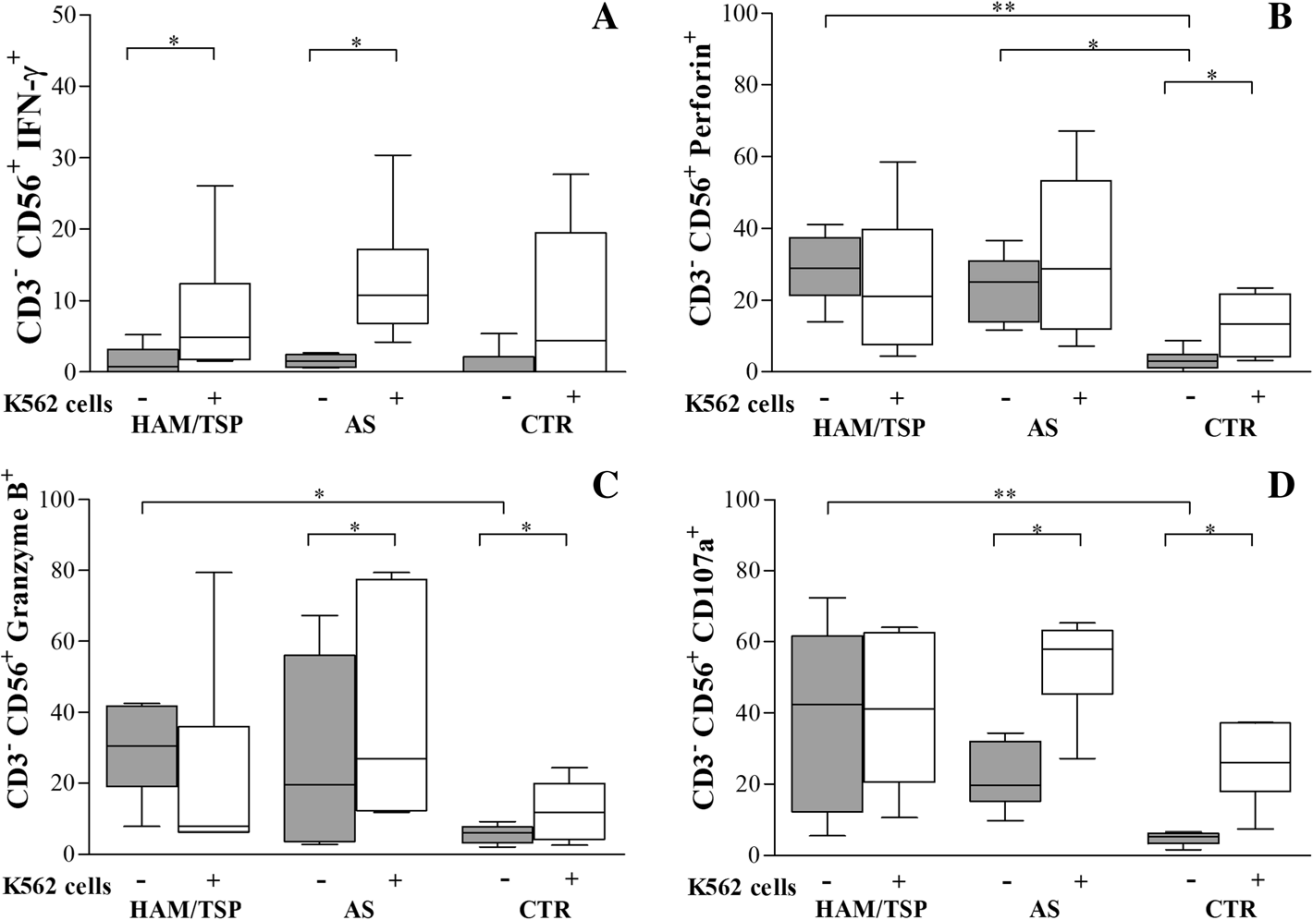
Cytotoxic activity of NK cells from HTLV-1 infected individuals against K562 target cells. HAM/TSP: HTLV-1 associated myelopathy/Tropical spastic paraparesis, *n* = 6; AS: asymptomatic individuals, n = 6; CTR: uninfected controls, *n* = 6. Peripheral blood mononuclear cells (PBMC) were cultured in presence or absence of K562 cells (1:1) for 6 h. **a** expression of IFN-γ, (**b**) expression of perforin, (**c**) expression of granzyme B, (**d**) expression of CD107a. Data are expressed as median and interquartile interval range. Differences considered significant when P ≤ 0.05. Kruskal-Wallis test, followed by Dunn’s post-test

**Fig. 4:**
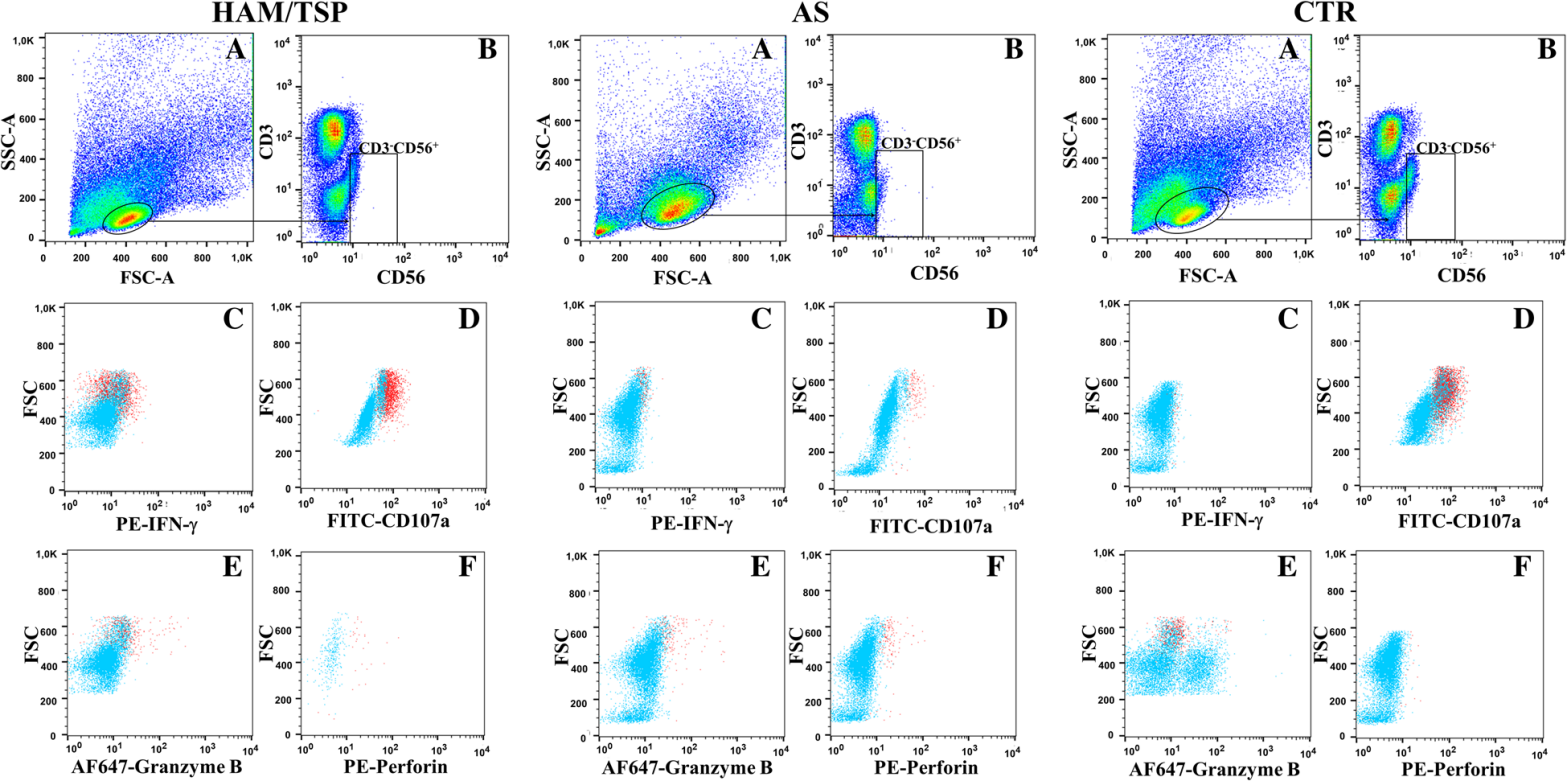
Representative flow plots for cytokine staining and cytotoxic molecules of NK cells. Lymphocyte gate (A). Selected NK cells (CD3^−^CD56^+^) (B). Representative flow plots showing expression of IFN-γ (C), (CD107a (D), granzyme B (E) and perforin (F) by NK cells from one representative individual with HAM/TSP, an asymptomatic HTLV-1-infected individual (AS) and one uninfected control (CTR), cultured in the presence of K562 for 6 h. Pink dot plots represent specific–stained population and blue dot plots represent background fluorescence observed with matched isotype control. Results reflect one representative experiment out of six independent experiments performed

To determine the functional significance of the present phenotypic findings, we investigated the intracellular expression of IFN-γ by NK cells, as well as the cytotoxic markers perforin, granzyme B and CD107a. While no differences were seen in IFN-γ expression in the absence of stimulation with K562 target cells, this functional marker was found to be significantly upregulated in both HAM/TSP (*p* = 0.03) and AS (*p* = 0.03) individuals groups compared to CTR (Fig. 3a) following stimulation. The percentage of unstimulated NK cells expressing perforin (Fig. 3b) and granzyme B (Fig. 3c) was significantly higher among HTLV-1-infected individuals (HAM/TSP and AS) compared to CTR (*p* = 0.003 and *p* = 0.04, respectively). In response to stimulation with K562 target cells, perforin expression was found to be increased only in CTR NK cells, while granzyme B expression was higher in NK cells from the AS and CTR groups. The evaluation of the degranulation capacity of NK cells revealed elevated levels of CD107a in HLTV-1-infected individuals compared to CTR in the absence of stimulation by target cells, and even higher levels in HAM/TSP (*p* = 0.03) than in AS (Fig. 3d). Moreover, the frequency of CD107a^+^ NK cells was higher in AS and CTR groups following stimulation by K562 target cells, yet no significant changes were observed in the frequency of these cells in the HAM/TSP group regardless of stimulation. Representative flow plots showing relative NK cell expression of CD107a, perforin, granzyme B and IFN-γ are shown in Fig. 4.

### Effect of Nkp30 receptor blockage on cytotoxic marker and IFN-γ expression by NK cells

**Fig. 5:**
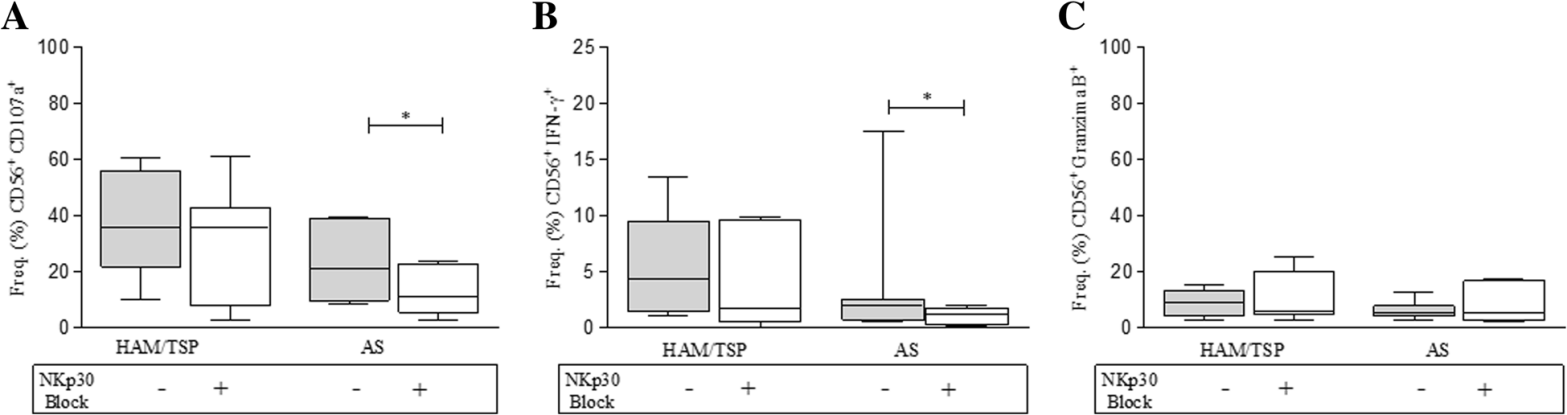
Cytotoxic activity of NK cells from HTLV-1 infected individuals against K562 target in the presence or absence of anti-NKp30 monoclonal antibody. Peripheral blood mononuclear cells (PBMC) were cultured in the presence of K562 cells (1:1), and in the presence or absence of NKp30 monoclonal antibody (4 μg/ml), for 6 h. **a** Expression of CD107a, (**b**) expression of IFN-γ, (**c**) expression of granzyme B. In controls, cytotoxic activity (CD107a) was reduced by 36% in the presence of anti-NKp30 antibody. Data are expressed as medians and interquartile interval range. HTLV-1 associated myelopathy/Tropical spastic paraparesis (HAM/TSP), n = 6; AS: asymptomatic individuals, n = 6. Differences were considered significant when P ≤ 0.05, Wilcoxon-test. *p = 0.01

To evaluate the relationship of NKp30 inhibition with respect to cytotoxicity, an assay using the anti-NKp30 monoclonal antibody was performed in an attempt to block its receptor (Fig. 5). While blockage of the NKp30 receptor did not alter cytotoxic activity (CD107a) or IFN-γ expression in the HAM/TSP group, cytotoxicity decreased by 48% (*P* = 0.01) and IFN-γ expression fell by 42% (*P* = 0.01) in the AS group. Similar results were also observed with respect to the degranulation markers analyzed after 18 h of culturing (data not shown). Of note, no correlations were found between HTLV-1 proviral load and any of the phenotypic or functional NK-cell markers investigated herein.

## Discussion

Few reports have described decreased cytotoxic activity in NK cells in HTLV-1 infection [18, 21], and none have attempted to evaluate cytotoxic function through the use of degranulation markers. The present study provides novel insight into the involvement of NK cells in the pathophysiology of HTLV-1. Specifically, we observed a decrease in the frequency of NK cells expressing the activating receptor NKp30 in individuals with a diagnosis of HAM/TSP compared to uninfected controls, as well as high degranulation activity in the absence of stimuli, as reflected by increased cytolytic (granzyme B and perforin) and degranulation marker expression. Moreover, NK cells from HAM/TSP individuals exhibited no increases in NK cells expressing degranulation markers (CD107a) or granzyme B following stimulation with K562 cells, as compared to AS and CTR groups. Additionally, blockage of the activating receptor NKp30 had no effect on the cytotoxic activity of NK-cells or IFN-γ expression in HAM/TSP individuals, in contrast to the decreased expression of these markers seen in asymptomatic carriers. These results indicate that NK cells from HTLV-1-infected individuals are in a state of continuous activation, especially the hypo-responsive NK cells found in HAM/TSP individuals. HTLV-1 infection is known to induce a potent activation of the immune system in both HAM/TSP and asymptomatic individuals [11, 12]. The spontaneous proliferation of T-cells and NK cells, increased expression of the activation markers HLA-DR, CD25 and CD45RO^+^, and increased proinflammatory cytokine production are all found to a greater extent in HTLV-1-infected individuals compared to uninfected controls [11, 12, 22, 23].

Additionally, the expression of TIM-3 and PD-1 was similar among groups, suggesting that exhaustion was not implicated in the hypo-responsiveness observed in NK cells from HAM/TSP individuals. However, it is possible that other persistent viral infections might induce cellular exhaustion, thereby leading to an impairment in effector function [24]. Indeed, SIV-infected non-human primate NK cells showed increased TIM-3 expression and failed to lyse target cells [25]. In addition, increased TIM-3 and PD-1 were also described in NK cells from individuals with hepatitis and cytomegalovirus [26].

In this study, we observed that levels of NKp30 decreased significantly in HAM/TSP patients. The activating NKp30 receptor has also been associated with increased NK cell efficiency in the lysing of tumor cells. In the context of other chronic viral infections, lower NKp30 expression was found in HPV-associated cervical cancer [27], AIDS [28] and HCV-infected individuals with cirrhosis [29]. In HIV-infected individuals, reduced NKp30 expression was observed in CD56^dim^ and CD56^neg^ NK cell subsets, although this was not determined to be of prognostic value [30]. Similarly, in acute viral infection, such as dengue virus type 2, NK cells expressed significantly lower levels of NKp30 compared to healthy individuals [31]. Accordingly, reductions in NKp30 may be indicative of alterations in innate immune response, as reflected by its occurrence in the context of severe manifestations of chronic viral infection, e.g. individuals with HAM/TSP.

Distinct isoforms of NKp30 may impact NK function. To date, three splice variants of NKp30 have been identified: NKp30C, an immunosuppressive isoform, as well as the activating isoforms NKp30A and B, which have been reported to affect NK cell function and may be correlated with the clinical outcome of gastrointestinal tumors (GIST). Low NKp30B/C ratios have been observed in response to higher transcription levels of isoform C, while a low NKp30A/C ratio was attributed to diminished isoform B expression; both of these findings suggest that differing ratios of the NKp30 isoforms may influence the outcome of GIST [32]. Furthermore, surface molecules BAT-3 and B7-H6 have been described as NKp30 cellular ligands. Despite the fact that Semeraro and colleges presented evidence regarding the clinical impact of NKp30 and its ligand B7-H6 [33] in patients with high risk neuroblastoma [34], no studies have clarified this association in the outcome of viral infections.

It has been previously suggested that the sensitivity of HTLV-1-positive cell lines to NK-mediated cell lysis was inversely correlated with tumorigenicity in an SCID model [35], implying that NK cells may prevent tumor induction and/or development in vivo. The efficiency of NK cells as a defense mechanism remains a topic of debate in chronic infections, such as HTLV-1. Hanon et al. (2000) did not observe significant reductions in CD4^+^ T-cells infected by HTLV-1 in NK-depleted cell cultures as compared to CD8^+^ T-lymphocytes, suggesting that NK cells may play a limited role in the control of HTLV-1 infection [13]. These conflicting results might also be reflective of major inconsistencies among *experimental* models. Regardless, further study is required to determine whether NK cells represent an efficient defense mechanism, especially in the context of HTLV-1.

The present study found high rates of spontaneous degranulation, which resulted in the elevated production of granzyme B and perforin, as well as IFN-γ expression, in NK cells from HTLV-1-infected individuals. Unexpectedly, NK cells from HAM/TSP subjects became hypo-functional in response to stimulation with K562 target cells, in spite of the elevated IFN-γ production typically seen in HTLV-1 infection [36, 37]. Reduced cytotoxic activity in HAM/TSP individuals was previously associated with a lower frequency of NK cells expressing CD16^+^ and CD11b^+^ [18, 21, 38], which might indicate the possible role of antibody-dependent cellular cytotoxicity mediated by NK cells.

A clear association between HAM/TSP diagnosis and high HTLV-1 proviral load has been observed in several studies conducted in Japan, Martinique, Brazil, United Kingdom and Iran [7, 8, 39–41]. Herein HTLV-1 proviral load was also consistently higher in HAM/TSP patients compared with AS individuals, however no correlations were found between HTLV-1 proviral load and any of the markers evaluated. The absence of correlations could be due to the relatively small number of individuals evaluated. However, while our data do not provide evidence that proviral load is associated with another NK marker (not tested in this study), we can highlight that NKp30 expression was, for the first time, found to discriminate asymptomatic individuals from HAM/TSP patients.

A limitation of the present study was that the NK cells evaluated were derived from total PBMCs instead of taking into account a purified population. However, as this subset constitutes a very small portion of PBMCs, the purification of these cells would require much larger amounts of blood to be drawn from patients, which was infeasible. Another important limitation was that no correlation could be established between the clinical outcomes of HTLV-1-infected patients and NK cell marker expression, which was likely a result of the limited number of studied individuals and the highly variable expression seen in the markers evaluated.

## Conclusions

In summary, unstimulated NK cells from HAM/TSP patients presented decreased expression of the NKp30 receptor and higher levels of cytolytic markers in comparison to asymptomatic individuals and uninfected controls. Moreover, NK cells from HAM/TSP individuals were found to be hypo-responsive following stimulation with target cells or blockage of the NKp30 receptor. These findings seem to suggest that decreases in the expression of NKp30 could influence the functional capacity of NK cells in subjects with HAM/TSP. Further studies should be conducted to comprehensively evaluate the role of interactions between activating/inhibiting receptors and their ligands with respect to cytotoxic response.

## Abbreviations

AS: Asymptomatic HTLV-1
ATLL: Adult T-cell leukemia/lymphoma
CHTLV: Integrative and Multidisciplinary Center for HTLV (a center that provides care for patients infected with the virus)
HAM/TSP: HTLV-1 Associated Myelopathy / Tropical Spastic Paraparesis
HTLV-1: Human T-lymphotropic virus type 1
NK: Natural killer
